# Adaptation to a low carbohydrate high fat diet is rapid but impairs endurance exercise metabolism and performance despite enhanced glycogen availability

**DOI:** 10.1113/JP280221

**Published:** 2020-08-19

**Authors:** Louise M. Burke, Jamie Whitfield, Ida A. Heikura, Megan L. R. Ross, Nicolin Tee, Sara F. Forbes, Rebecca Hall, Alannah K. A. McKay, Alice M. Wallett, Avish P. Sharma

**Affiliations:** ^1^ Exercise and Nutrition Research Program Mary MacKillop Institute for Health Research Australian Catholic University Melbourne Australia; ^2^ Australian Institute of Sport Canberra Australia; ^3^ School of Human Sciences (Exercise and Sport Science) University of Western Australia Crawley Australia; ^4^ Western Australian Institute of Sport Mt Claremont Western Australia Australia; ^5^ University of Canberra Research Institute for Sport and Exercise Canberra Australia; ^6^ Griffith Sports Physiology and Performance School of Allied Health Sciences Griffith University Southport Australia

**Keywords:** athletic performance, ketogenic diet, sports nutrition

## Abstract

**Key points:**

- Brief (5–6 days) adaptation to a low carbohydrate high fat diet in elite athletes increased exercise fat oxidation to rates previously observed with medium (3–4 weeks) or chronic (>12 months) adherence to this diet, with metabolic changes being washed out in a similar time frame.
- Increased fat utilisation during exercise was associated with a 5–8% increase in oxygen cost at speeds related to Olympic Programme races.
- Acute restoration of endogenous carbohydrate (CHO) availability (24 h high CHO diet, pre‐race CHO) only partially restored substrate utilisation during a race warm‐up. Fat oxidation continued to be elevated above baseline values although it was lower than achieved by 5–6 days’ keto adaptation; CHO oxidation only reached 61% and 78% of values previously seen at exercise intensities related to race events.
- Acute restoration of CHO availability failed to overturn the impairment of high‐intensity endurance performance previously associated with low carbohydrate high fat adaptation, potentially due to the blunted capacity for CHO oxidation.

**Abstract:**

We investigated substrate utilisation during exercise after brief (5–6 days) adaptation to a ketogenic low‐carbohydrate (CHO), high‐fat (LCHF) diet and similar washout period. Thirteen world‐class male race walkers completed economy testing, 25 km training and a 10,000 m race (Baseline), with high CHO availability (HCHO), repeating this (Adaptation) after 5–6 days’ LCHF (*n* = 7; CHO: <50 g day^−1^, protein: 2.2 g kg^−1^ day^−1^; 80% fat) or HCHO (*n* = 6; CHO: 9.7 g kg^−1^ day^−1^; protein: 2.2 g kg^−1^ day^−1^) diet. An Adaptation race was undertaken after 24 h HCHO and pre‐race CHO (2 g kg^−1^) diet, identical to the Baseline race. Substantial (>200%) increases in exercise fat oxidation occurred in the LCHF Adaptation economy and 25 km tests, reaching mean rates of ∼1.43 g min^−1^. However, relative ${\dot{V}}_{O_{2}}$ (ml min^−1^ kg^−1^) was higher (*P *< 0.0001), by ∼8% and 5% at speeds related to 50 km and 20 km events. During Adaptation race warm‐up in the LCHF group, rates of fat and CHO oxidation at these speeds were decreased and increased, respectively (*P *< 0.001), compared with the previous day, but were not restored to Baseline values. Performance changes differed between groups (*P *= 0.009), with all HCHO athletes improving in the Adaptation race (5.7 (5.6)%), while 6/7 LCHF athletes were slower (2.2 (3.4)%). Substrate utilisation returned to Baseline values after 5–6 days of HCHO diet. In summary, robust changes in exercise substrate use occurred in 5–6 days of extreme changes in CHO intake. However, adaptation to a LCHF diet plus acute restoration of endogenous CHO availability failed to restore high‐intensity endurance performance, with CHO oxidation rates remaining blunted.

## Key points

- Brief (5–6 days) adaptation to a low carbohydrate high fat diet in elite athletes increased exercise fat oxidation to rates previously observed with medium (3–4 weeks) or chronic (>12 months) adherence to this diet, with metabolic changes being washed out in a similar time frame.
- Increased fat utilisation during exercise was associated with a 5–8% increase in oxygen cost at speeds related to Olympic Programme races.
- Acute restoration of endogenous carbohydrate (CHO) availability (24 h high CHO diet, pre‐race CHO) only partially restored substrate utilisation during a race warm‐up. Fat oxidation continued to be elevated above baseline values although it was lower than achieved by 5–6 days’ keto adaptation; CHO oxidation only reached 61% and 78% of values previously seen at exercise intensities related to race events.
- Acute restoration of CHO availability failed to overturn the impairment of high‐intensity endurance performance previously associated with low carbohydrate high fat adaptation, potentially due to the blunted capacity for CHO oxidation.

## Introduction

According to current sports nutrition guidelines, the performance of sustained high‐intensity endurance sports is best supported by conditions of high carbohydrate (CHO) availability (Thomas *et al*. 2016), defined as matching the finite body CHO stores to event‐specific muscle and central nervous system fuel needs (Burke *et al*. 2018 *a*). High CHO availability (HCHO) can be achieved via targeted dietary CHO intake over the 24–36 h prior to the race to normalise or super‐compensate muscle glycogen concentrations (Burke *et al*. 2011), supplemented by a pre‐race CHO‐rich meal to restore liver glycogen content and provide ongoing release of glucose from the gut within the race (Coyle, 1991). In events lasting longer than 90 min, the provision of additional CHO substrate from foods/fluids consumed during exercise becomes more important (Stellingwerff & Cox, 2014). Contemporary views around everyday nutritional support for the high‐volume endurance training necessary to prepare for such events also promote HCHO availability, at least for key workouts in which high‐intensity/quality performance or race simulation is desired (Burke *et al*. 2011, 2018a). Yet, competitive endurance athletes do not always achieve these goals, for reasons that are either accidental or intentional (Heikura *et al*. 2018).

A deliberate contrast to these guidelines is the revived interest in adaptation to low CHO, high fat (LCHF) diets, due to observations that athletes can achieve substantial increases to their already enhanced capacity for oxidising fat during exercise, including an increase in the exercise intensity at which maximal rates of fat oxidation occur (for review, see Burke, 2021). The specific multi‐system physiological responses to keto adaptation are contentious, as are the timelines required to achieve them (Lindseth, 2017; Burke *et al*. 2020). However, in the context of exercise performance, the key adaptations appear to be increased delivery, uptake and subsequent oxidation of free fatty acids (for review see Burke, 2021). The current model of interest, involving chronic adherence to a ketogenic version of the diet (<50 g day^−1^ CHO, 15–20% energy from protein and 75–80% fat) has been enthusiastically proposed via social, lay and peer‐reviewed media to provide universal benefits to the performance of endurance and ultra‐endurance sports (Noakes *et al*. 2014; Burke, 2015; Volek *et al*. 2015). However, rigorously controlled studies from our group have reported detriments to the real‐life performances of high‐intensity endurance events in elite athletes following 3–4 weeks of a LCHF diet, in comparison to training/racing with HCHO availability (Burke *et al*. 2017). Indeed, substantial increases in fat utilisation are associated with an increased oxygen cost of exercise, particularly in high‐intensity (>70% ${\dot{V}}_{O_{2}\mathrm{peak}}$) domains (Burke *et al*. 2017; Shaw *et al*. 2019, 2020). Whereas the aerobic reserve can accommodate this trade‐off during moderate intensity exercise, oxygen availability may become a limiting factor for energy production at higher intensities, contributing to a reduction in performance.

Although the concept of ‘tapping into unlimited fuel stores’ remains an intriguing proposition for gaining a competitive advantage in endurance/ultra‐endurance sport, it presupposes that adequate fuel is available for event demands across the characteristic exercise intensities as well as duration. Our continued investigation of the ketogenic LCHF diet has focused on better understanding the mechanisms of adaptation and, potentially, overcoming the inherent limitations of reliance on fat utilisation at the higher intensities of exercise that are critical to the success of higher level competitors (Burke, 2021). One philosophy which attempts to ‘gain the best of both worlds’ involves periodising LCHF with HCHO availability, e.g. acute pre‐race restoration of glycogen in keto adapted athletes. We have previously shown in well‐trained athletes that robust increases in fat oxidation occur in as little as 5–6 days of a non‐ketogenic LCHF diet (Burke *et al*. 2000; Carey *et al*. 2001, 2002); however, this has not been established with elite athletes and a ketogenic LCHF diet. Indeed, such findings would contradict a popularly held view, around which our studies have received vocal social media criticism, that ‘keto adaptation’ requires several months to achieve (Burke, 2021; Burke *et al*. 2020).

Accordingly, we developed a protocol to investigate the metabolic and performance effects of 5–6 days’ exposure to a ketogenic LCHF diet, followed by restoration of muscle glycogen content, in a cohort of elite race walkers in a real‐life race. In addition to changing the duration of keto adaptation compared with our previous studies, we altered the feeding protocol to preserve energy availability by altering daily energy intake according to the training energy expenditure. The study was designed to address the following questions: (1) Can very short‐term exposure to an established ketogenic LCHF diet achieve robust and substantial changes in capacity for fat oxidation during exercise at intensities of interest to competitive sport? (2) Can the previously found decrement in race performance involving sustained high‐intensity exercise associated with such exposure to LCHF be ‘rescued’ by a 24 h period of high CHO availability? And (3) can the changes to metabolism and substrate utilisation during exercise achieved by short‐term adaptation to LCHF be reversed by restoration of HCHO over a similar time frame? We hypothesised that substantial changes in substrate utilisation during exercise would both occur and reverse in 5–6 days, and that negative effects on performance due to LCHF‐associated reliance on fat utilisation would be overturned by a 1‐day protocol known to at least normalise muscle glycogen.

## Methods

### Ethical approval

This study was registered with the Australian New Zealand Clinical Trials Registry (ACTRN12618001974291p). It conformed to the standards set by the *Declaration of Helsinki* and was approved by the Human Research Ethics Committee of the Australian Institute of Sport (no. 20181203). Comprehensive details of the study protocol were explained orally and in writing prior to athletes providing their written informed consent.

### Overview of study design

**Figure 1:**
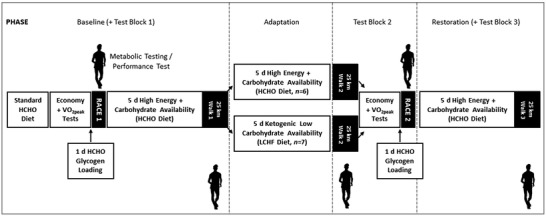
Overview of 3 week training camp, testing blocks and dietary intervention

This parallel‐groups designed study was conducted within a training camp that represented baseline preparation for the 2019 World Athletics (WA; formerly International Association of Athletics Federations) race‐walking season. The study took place at the Australian Institute of Sport (AIS) with athletes living in athlete residences and being supervised at all meals and training sessions. The study consisted of a 4‐week structured training block divided into three phases (see Fig. 1). Briefly, upon arriving at the training camp, athletes undertook tests (Test Block 1, Baseline) to identify baseline characteristics around aerobic capacity (${\dot{V}}_{O_{2}\mathrm{peak}}$ and exercise economy tests), performance (WA sanctioned 10,000 m race walk event, Race 1). This period involved a standardised diet of high energy and CHO availability during the first testing protocols, followed by a further 5‐day period with individualised achievement of the same high energy/CHO availability (HCHO, see below) to support structured training and prepare for the Baseline 25 km test. After the completion of this test, athletes were divided into two groups to commence a 5‐day dietary intervention (Adaptation) before repeating all testing elements. Test Block 2 (Adaptation) included a second 25 km walk test and economy/${\dot{V}}_{O_{2}\mathrm{peak}}$ tests while following the intervention diets (HCHO or ketogenic LCHF) before moving to the same standardised 24 h pre‐race dietary preparation undertaken for Race 1 (HCHO availability) prior to a second performance test (WA sanctioned 10,000 m race walk event, Race 2). To determine if the effects of the dietary interventions could be reversed, all athletes completed Phase 3, which involved 5 days on the HCHO diet and a third 25 km walk test (Test Block 3, Restoration). Since the absolute duration of each intervention involved 5 days of dietary intake and training, plus the period of the testing block (2–3 days) , we have simplified the study report to describe the intervention as a 6‐day period.

### Athletes

Fourteen male race walkers with international race experience were recruited for the study to fulfil our intention, based on resources and experience from previous studies of sufficient power to detect differences in metabolic variables when rigorous control of similar study intervention and testing protocols was implemented (Burke *et al*. 2017). Specific sample size estimation was calculated for performance measures using G Power software (Version 3.1, Bonn University, Bonn, Germany) based on our previous research in which the impact of LCHF on a 10,000 m race performance was investigated in a single camp (Burke *et al*. 2020). Based on such data, a sample size of seven athletes per group was considered appropriate (*n* = 7, critical *t* = 2.179; expected power = 0.939; *P *< 0.05). One athlete experienced visa delays and was unable to attend, leaving us with 13 athletes for this data set. The cohort ranged from world class athletes (e.g. Olympians, World Championship and WA Race Walking Team Championships medallists, national record holders) to highly trained athletes (e.g. training partners of world class athletes). Specifically, all athletes had competed internationally for their country during their career, and 11 of the 13 athletes included in this study were selected for at least one of the major events in the previous two seasons (2017 WA World Championships or the 2018 WA Race Walking Team Championships). Athletes were educated about the benefits and limitations of the dietary interventions and asked to nominate their preference for, or non‐acceptance of, each intervention, as described previously (Burke *et al*. 2017). We were able to allocate race walkers to a preferred dietary condition while achieving suitable matching between groups based on age, body mass, aerobic capacity and personal best for the 20 km race walk (Table 1). Seven athletes were previous participants in our earlier studies (Burke *et al*. 2017, 2020), with one choosing to repeat the LCHF intervention.

**Table 1:** Characteristics of the elite race walkers who participated in this study

Characteristic	High energy/CHO availability (HCHO) (*n* = 6)	Ketogenic low CHO, high fat (LCHF) (*n* = 7)
Age (years)	32.7 (4.8)	28.3 (2.7)
Body mass (kg)	66.2 (7.8)	66.2 (8.1)
Baseline ${\dot{V}}_{O_{2}\mathrm{peak}}$ (ml kg^−1^ min^−1^)	63.85 (3.63)	67.70 (6.11)
Personal best in 10 km race walk (min:ss.00)	40:56.40 (1:08.58)	41:20.92 (2:17.03)
Personal best in 20 km race walk (h:min:ss)	1:23:40 (2:09)	1:21:55 (2:50)

Data are means (SD).

### Body composition and resting metabolic rate

Assessment of fat free mass (FFM) and resting metabolic rate (RMR) was undertaken during Test Block 1 for use in the calculation of energy intake based on energy availability (EA) calculations. FFM was determined after an overnight fast and rest via dual X‐ray absorptiometry (DXA) using a standardised protocol previously described (Nana *et al*. 2015) and an iDXA (GE Healthcare, Milwaukee, WI, USA) with image analysis (enCore v16, GE Healthcare). Resting metabolic rate was assessed according to the standardised outpatient protocol described in full elsewhere (Bone & Burke, 2018)

### Dietary intervention

All foods and fluids consumed during the study were provided and recorded by the research team, with menu construction and food preparation being undertaken by a professional chef, food service dietitians and sports dietitians. Meal plans were individually developed for each athlete as reported previously (Mirtschin *et al*. 2018), to integrate personal food preferences and nutrition requirements within the daily energy availability targets, planned energy expenditure of training and macronutrient goals. Daily checks of compliance to the training plan, dietary prescription and reporting requirements were undertaken, with food choices and portion sizes being adjusted at meals eaten in the later part of the day if actual training deviated from the plan.

Since a similar workload (training/testing protocols) was undertaken by each athlete in preparation for the first part of Test Block 1, we provided a standardised diet intended to provide high energy/CHO availability, based on our previous studies (Burke *et al*. 2017), of 56 kcal (225 kJ) kg^−1^ body mass (BM) and 8.5 g CHO kg^−1^ BM day^−1^ over this short time period. Once estimations of FFM and RMR were completed, provision of daily energy intake was then undertaken according to EA estimations. Our goal was to provide diets with EA of ∼40 kcal (168 kJ) kg^−1^ FFM day^−1^ where EA was defined and implemented according to the original principle of Loucks (2004) as:

Energy intake – (Energy cost of exercise; equal to net increase in energy expenditure above sedentary rates over the period of exercise)/FFM (kg)

This involved variation for each individual and each day according to the specific training load, including real‐time adjustments due to differences between planned and actual activities. The dietary treatments investigated in the present study are summarised below:

#### High‐energy, high‐CHO availability (HCHO)

The principle of this diet was to provide optimal CHO availability for all training sessions (Burke *et al*. 2018 *a*), with an adjustment from protocols in previous studies (Burke *et al*. 2017) of manipulating energy and CHO intake according to the fuel cost of training. Daily variability in energy intake from the baseline diet of 56 kcal (225 kJ) kg^−1^ BM and ∼8.5 g kg^−1^ BM CHO was calculated according to the EA equation, and achieved by manipulating CHO, protein and fat intake in a standardised ratio (65% CHO, 15% protein, 20% fat). CHO intake was spread across all meals, as well as snacks intended for consumption pre‐, during‐ and post‐training. Protein intake was targeted at 2–2.2 g kg^−1^ day^−1^.

#### Ketogenic low‐carbohydrate, high‐fat diet (LCHF)

This diet was matched to the (high) EA of HCHO (∼40 kcal kg^−1^ FFM day^−1^), with very restricted CHO intake (0.5 g kg^−1^ day^−1^ or <50 g day^−1^), matched protein intake (∼2–2.2 g kg^−1^ day^−1^) and the remaining (75–80%) energy as fat. This is similar to the LCHF diets implemented in our previous studies (Burke *et al*. 2017; Mirtschin *et al*. 2018) and was based on the dietary philosophies and meal plans of current LCHF thought‐leaders (Volek & Phinney 2012). The stricter achievement of individual and daily EA goals was reliant on adjustment of fat intake since both CHO and protein targets were fixed.

#### Test meal

Each of the test protocols (incremental testing of economy and ${\dot{V}}_{O_{2}\mathrm{peak}}$, the 10,000 m race, and the standardised 25 km race walking session) was preceded by a standardised test meal that varied only according to the intervention. The same CHO‐rich meal providing 2 g kg^−1^ CHO was consumed 2 h prior to the commencement of each of the test protocols in the Baseline and Restoration trials, and during the Adaptation Test Block in the case of the HCHO group. Meanwhile, the LCHF group received an energy‐matched meal that was high‐fat and low‐CHO on these occasions.

### Incremental testing (economy and ${\dot{V}}_{O_{2}\mathrm{peak}}$)

During Test Blocks 1 and 2 (Baseline and Adaptation), athletes completed an incremental exercise test to exhaustion on a custom‐built motorised treadmill (Australian Institute of Sport, Bruce, Australia) to determine exercise economy and ${\dot{V}}_{O_{2}\mathrm{peak}}$ while race walking, as previously reported (Burke *et al*. 2017). Briefly, walking economy was assessed during four submaximal stages, each lasting 4 min and increasing in speed by 1 km h^−1^ each stage. Starting speeds were selected at either 11 or 12 km h^−1^ based on each individual's capacity; 20 km personal best times were compared with the 2019 WA World Championship qualifying standard of 1 h 22 min 30 s, with athletes faster than this mark commencing at 12 km h^−1^ and increasing to 15 km h^−1^ at the final stage. The speeds of the second and fourth stage corresponded approximately to each individual athlete's walking pace for the 50 and 20 km race walk event, respectively. This test commenced 2 h following the intake of the standardised test meal as outlined above.

Each stage was followed by 1 min rest for the collection of capillary (fingertip) blood samples to assess blood lactate (Lactate Pro 2, Akray, Japan), ketone bodies (β‐hydroxybutyrate (βHB); FreeStyle Optium Neo, Abbott Diabetes Care, Doncaster, Victoria, Australia) and glucose (FreeStyle Optium Neo) concentrations, as well as ratings of perceived exertion (RPE, 6–20 Borg Scale). Heart rate (HR) was measured continuously throughout the test (Polar Heart Rate Monitor, Polar Electro, Kempele, Finland). Expired gas was collected and analysed using a custom‐built indirect calorimetry system described previously (Saunders *et al*. 2010), with the final 60 s of gas collected accepted as steady state and used to calculate respiratory exchange ratio (RER) and O_2_ uptake. A self‐selected warm‐up of 10 min duration preceded the test, which was maintained across trials.

Upon completion of the final submaximal walking stage, athletes rested for 5 min before completing a ramp (speed and then gradient) test to volitional fatigue. Treadmill speed was increased by 0.5 km h^−1^ every 30 s until the speed corresponding to the individual's final submaximal stage was reached (14 or 15 km h^−1^), with treadmill gradient increased by 0.5% every 30 s thereafter until exhaustion. Expired gas was collected and analysed throughout, maximal HR recorded, and capillary blood samples collected 1 min after completion.

### 10,000 m race

During the Baseline and Adaptation Test Blocks, athletes competed in a 10,000 m race held on a synthetic 400 m outdoor athletics track (Canberra, ACT, Australia). To provide an incentive for a maximal effort, prize money was awarded to place getters as well as those athletes who achieved the highest percentage of their 20 km walking personal best when the times of the two races were combined. Each race commenced at 09.00 h and was conducted under WA rules, which involved officiating by technical judges, invitation for participation by competitors external to the study, a feed zone allowing water intake on the outside lanes of the track in hot conditions, the pit‐lane rule for technique infringements and photo‐finish electronic timing. Photo‐finish timing was used to provide official race times. Capillary blood samples were collected immediately before the start of the race and as each competitor completed the race.

The goals of the race nutrition plan were to standardise the individualised competition practices of each participant. Therefore, each athlete repeated a similar training load during the 48 h prior to each race (economy/aerobic capacity test schedule and personal training) and consumed the same HCHO diet over the 24 h pre‐race period, as well as the consuming the same CHO‐rich (2 g kg BM^−1^) pre‐race meal 2 h prior to competition. The use of caffeine as a performance supplement by some participants was permitted under the following conditions: it was documented in pre‐race diaries, came from a reliable source (e.g. the same sports supplement) and was repeated in an identical protocol for both races. Due to the hot environmental conditions, the WA policy to allow a water station in an outside lane was implemented for both races. The athletes were able to choose freely to deviate from the inside track and obtain water or ice for consumption and cooling during the race; all made use of this resource.

Warm‐up for the 10,000 m race was undertaken according to our previous studies (Burke *et al*. 2017), whereby each athlete chose his own protocol, based on their real‐life experiences, and replicated this for each race. In the case of athletes allocated to the LCHF diet, the warm‐up was commenced in the laboratory to allow the impact of the 1‐day CHO restoration protocol on substrate utilisation and exercise economy during exercise to be measured. Between 90 and 45 min pre‐race (standardised for each athlete across races), these athletes completed a two‐stage incremental exercise test involving a 2 min warm‐up at 10 km h^−1^ and two 4‐min stages (separated by 1 min at rest) at speeds corresponding to 50 and 20 km race walk events (i.e. 12 and 14 or 13 and 15 km h^−1^ depending on ability). HR was measured continuously during the test, and expired gas was collected and analysed, with the final 60 s of gas collected accepted as steady state and used to calculate RER and O_2_ uptake. Capillary blood samples were collected prior to warm‐up, and immediately after each stage to determine blood lactate, glucose and ketone concentration. Following completion of the test, athletes transferred to the athletics track (∼500 m walk) and completed the remainder of their race preparations, which were recorded and replicated across trials.

### Standardised 25 km long walk

At the end of each of the test blocks, athletes completed a 25 km walk, 2 h after consuming a standardised meal that met the conditions of their dietary intervention. The training session was conducted as a hybrid laboratory‐field test, with 0–1, 6–7, 12–13, 18–19 and 24–25 km being undertaken on a treadmill in the laboratory and the remainder of the walk completed outdoors on a loop course (∼5 km) which included two aid stations to allow nutrition support to be received every ∼2 km as occurs in WA events. Athletes completed the treadmill portions of the walk at the speed corresponding to the second stage of the submaximal walking test (12 or 13 km h^−1^), which approximated their 50 km race pace. Each negotiated an individualised but similar pace target (∼12 km h^−1^) for the outside portion of the walk with the goal that each athlete would adhere to this pace for all three of his walks. Expired gas was collected during each treadmill segment for assessment of RER to determine rates of substrate oxidation and O_2_ uptake. Capillary blood was collected for measurement of glucose, lactate and ketone concentrations immediately prior to beginning the session, and upon completion of each treadmill segment, using the protocols previously documented. HR and RPE were assessed at the end of each treadmill section.

The standardised pre‐exercise meal (previously described) was consumed 2 h prior to the commencement of the session. During the walk, handlers provided fluids and sports food choices that had previously been discussed with each participant to mimic race feeding practices. Athletes were offered sports gels and water to achieve an hourly intake of ∼600 ml of fluid and ∼60 g CHO in the Baseline and Restoration (both groups) and Adaptation (HCHO group). Meanwhile, in the LCHF Adaptation trial, athletes received non‐caloric fluid (electrolyte‐supplemented water) and LCHF cookies to match the energy intake from their other trial.

### Blood sampling and serum free fatty acid analysis

Prior to (fasted and post‐prandial pre‐exercise, ‘0 km’), during (1 km and 13 km), and immediately post (25 km) the 25 km‐long walk, blood was collected via an indwelling forearm cannula into 2.5 ml Vacuette SST tubes (Greiner Bio‐One, Kremsmünster, Austria), allowed to clot for 30 min then centrifuged at 1500 *g*, 4°C, 10 min. The collected serum was frozen and later analysed for free fatty acid (FFA) concentration using a colorimetric assay according to the manufacturer's instructions (NEFA‐c kit, Novachem Pty Ltd, Heidelberg West, Victoria, Australia).

### Calculation of respiratory exchange ratio and substrate oxidation data

Respiratory exchange ratio was calculated from steady‐state expired gases collected over 1 min periods during the economy test and maximal aerobic capacity (${\dot{V}}_{O_{2}\mathrm{peak}}$) protocol. Rates of CHO and fat oxidation (g min^−1^) were calculated from ${\dot{V}}_{CO_{2}}$ and ${\dot{V}}_{O_{2}}$ values using non‐protein RER values (Peronnet & Massicotte, 1991). These equations are based on the premise that ${\dot{V}}_{O_{2}}$ and ${\dot{V}}_{CO_{2}}$ accurately reflect tissue O_2_ consumption and CO_2_ production, and that indirect calorimetry is a valid method for quantifying rates of substrate oxidation in well‐trained athletes during strenuous exercise of up to ∼85% of ${\dot{V}}_{O_{2}\mathrm{peak}}$ (Romijn *et al*. 1993). We did not correct our calculations for the contribution of ketone oxidation to substrate use in order to contextualise our findings with previous work performed by our group (Stellingwerff *et al*. 2006; Burke *et al*. 2017) as well as other reports of substrate utilisation in ultra‐endurance athletes who chronically consume LCHF diets (Volek *et al*. 2016; Webster *et al*. 2016). However, we acknowledge that there may be a small (but systematic) error in the use of conventional equations to calculate fat and CHO oxidation from gas exchange information (Frayn, 1983).

### Training

During each of the harmonisation and intervention periods, athletes were required to complete a 25–40 km‐long walk, interval training session (8–12 × 1 km on a 6 min cycle completed on a standard 400 m athletics track) and tempo hill session (14 km with ∼450 m elevation gain). The remaining training consisted of low intensity walking sessions (6–12 km each), and a strength training session. The daily energy cost of training (exercise energy expenditure; EEE), which represented the additional energy cost attributed to exercise during a training/test session period rather than the total energy expenditure during the period (Burke *et al*. 2018 *b*), was calculated by subtracting resting metabolic rate from session energy expenditure.

### Statistical analyses

**Figure 2:**
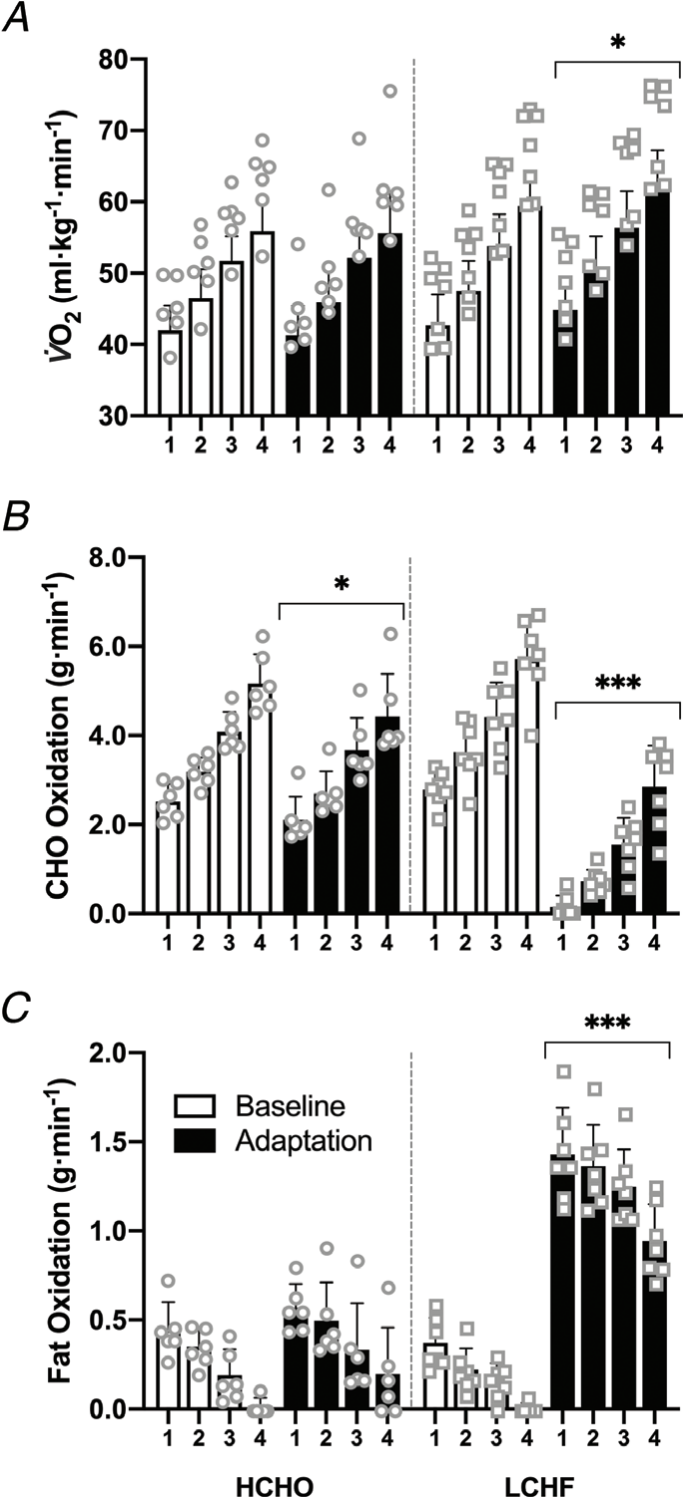
Oxygen uptake (*A*) and substrate utilisation (carbohydrate (CHO) (*B*) and fat oxidation (*C*)) during four‐stage economy test performed prior to and following adaptation to either a low CHO high fat (LCHF, *n* = 7) or high CHO (HCHO, *n* = 6) diet Data are means ± SD. Significant differences within group relative to Baseline: ^*^ *P *< 0.05, ^***^ *P *< 0.0001.

**Figure 3:**
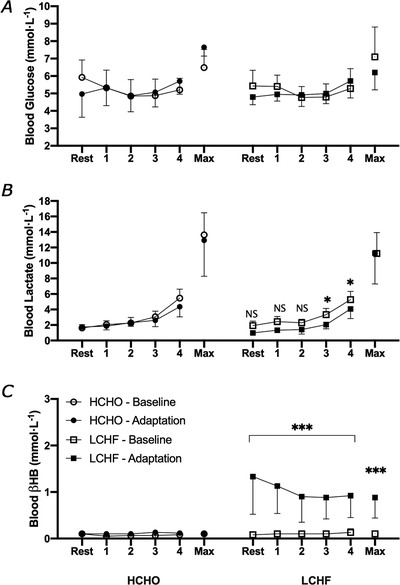
Blood metabolite concentrations Blood glucose (mmol L^−1^) (*A*), blood lactate (mmol L^−1^) (*B*) and blood β‐hydroxybutyrate (mmol L^−1^) (*C*) at rest, during four‐stage graded economy test and maximal aerobic capacity test (Max) prior to and following adaptation to either a low CHO high fat (LCHF, *n* = 6) or high CHO (HCHO, *n* = 6) diet. Data are means ± SD. Significant differences within group relative to Baseline: ^*^ *P *< 0.05, ^***^ *P *< 0.0001.

**Figure 4:**
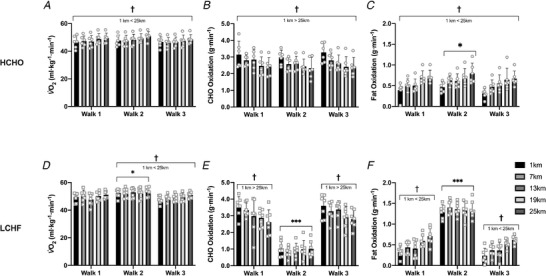
Oxygen uptake (*A* and *D*) and rates of carbohydrate (CHO; *B* and *E*) and fat (*C* and *E*) oxidation during standardised 25 km long walk performed at baseline (Walk 1), following adaptation to either a low CHO high fat (LCHF, *n* = 7) or high carbohydrate (HCHO, *n* = 6) diet (Walk 2) and restoration where all athletes consumed a HCHO diet (Walk 3) Data are mean ± SD. Significant change over the 25 km walking session (1 km < 25 km): †*P *< 0.01. Significantly different than Walk 1 and Walk 3: ^*^ *P *< 0.05, ^***^ *P *< 0.0001.

**Figure 5:**
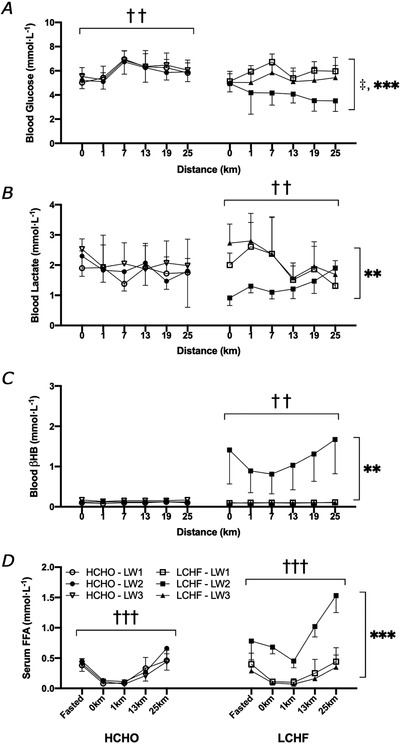
Blood metabolite concentrations Blood glucose (mmol L^−1^) (*A*), blood lactate (mmol L^−1^) (*B*), blood β‐hydroxybutyrate (mmol L^−1^) (*C*) and serum free fatty acids (mmol L^−1^) (*D*) at rest (0 km as well as fasted for FFA), and throughout standardized 25 km‐long walk performed at baseline (Walk 1), following adaptation to either a low CHO high fat (LCHF, *n* = 7) or high carbohydrate (HCHO, *n* = 6) diet (Walk 2) and restoration where all athletes consumed a HCHO diet (Walk 3). Data are means ± SD. Significant change over the 25 km walking session: ††*P *< 0.001, †††*P *< 0.0001. Significant differences between Walk 1 and Walk 3: ‡*P *< 0.05. Significant differences between Walks 1 and 3 compared to Walk 2: ^**^ *P *< 0.001, ^***^ *P *< 0.0001.

**Figure 6:**
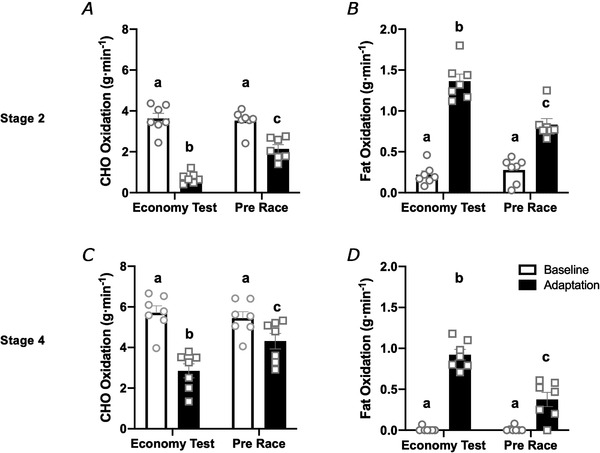
Substrate utilisation (carbohydrate (CHO) (*A* and *C*) and fat oxidation (*B* and *D*)) at stages representing 50 km (Stage 2) and 20 km (Stage 4) race speeds during economy tests Tests were performed prior to and following adaptation a low CHO high fat (LCHF, *n* = 7) diet as well as immediately prior to both Race 1 and Race 2. Both pre‐race tests were performed with a 1‐day HCHO diet to restore skeletal muscle glycogen content. Data are means ± SD, with results of individual athletes being shown by the circles (Baseline) and squares (Adaptation). Bars sharing a letter are not statistically different from one another (*P *< 0.05).

**Figure 7:**
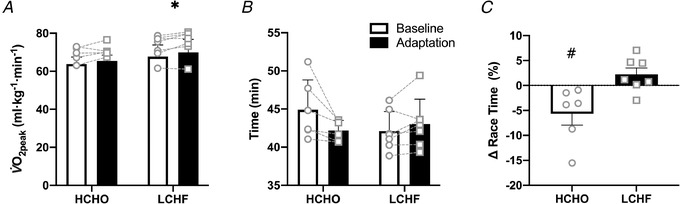
Relative maximum oxygen uptake (*A*), 10,000 m race performance (*B*) and performance changes between the two races (*C*) prior to and following adaptation to either a low CHO high fat (LCHF, *n* = 7) or high CHO (HCHO, *n* = 6) diet Data are means ± SD with results of individual being shown by the circles (Baseline) and squares (Adaptation). Significant differences within group relative to Baseline: ^*^ *P *< 0.05. Significant difference in performance changes between groups: †*P *= 0.009.

Results are expressed as means (SD). Data from dietary intake, economy tests, long walks and race performances (Figs 2, 3, 4, 5, 6, Tables 2, 3) were analysed using a two‐way analysis of variance (ANOVA) within dietary groups. If significance was detected, a Bonferroni *post hoc* test was applied. Student's *t* test was used to analyse differences between subject groups (Table 1), and performances in Baseline and Adaptation races (Fig. 7). Significance was set at *P *< 0.05 (‘NS’ indicates not significant). All statistical analyses were performed, and graphs were produced using GraphPad Prism (version 8.3.1, GraphPad Software Inc., La Jolla, CA, USA). All data collected in this study are presented in the figures and tables; raw data are available on request to the corresponding author.

**Table 2:** Dietary intake during 5–6 days’ adaptation to ketogenic LCHF diet and 5–6 days’ dietary restoration plus control diet in elite race walkers undertaking endurance training

	HCHO (*n* = 6)	LCHF (*n* = 7)
Baseline period: both groups – HCHO diet		
Energy intake (kJ day^−1^)	16565 (3039)	15762 (1997)^**,‡‡^
Energy intake (kJ kg day^−1^)	253 (27)	242 (13)^***^ ^,^ ^‡‡^
CHO (g day^−1^)	647 (122)	614 (78)^***^
CHO (g kg day^−1^)	9.9 (1.1)	9.4 (0.5)^***^
CHO (E%)	65 (0.4)	65 (0.4)^***^
Protein (g day^−1^)	151 (28)^‡^	144 (18)
Protein (g kg day^−1^)	2.3 (0.2)^‡^	2.2 (0.1)
Protein (E%)	15 (0.4)	15 (0.4)
Fat (g day^−1^)	88 (16)	84 (11)^***,‡‡^
Fat (g kg day^−1^)	1.3 (0.1)	1.3 (0.1)^***,‡‡^
Fat (E%)	20 (0.2)	20 (0.3)^***^
EEE (kcal day^−1^)	1633 (763)	1369 (259)^**^
EA (kcal kg FFM day^−1^)	39 (4)	41 (10)
Adaptation period: LCHF group – LCHF diet		
Energy intake (kJ day^−1^)	16116 (2922)	14769 (1750)^†^
Energy intake (kJ kg day^−1^)	246 (25)	227 (14)^†,§^
CHO (g day^−1^)	634 (114)^§§§^	35 (3)^†††^
CHO (g kg day^−1^)	9.7 (1.0)^§§§^	0.5 (0.0)^†††^
CHO (E%)	66 (0.3)^§§§^	4 (0.2)^†††^
Protein (g day^−1^)	144 (27)	145 (17)
Protein (g kg day^−1^)	2.2 (0.2)	2.2 (0.0)
Protein (E%)	15 (0.1)^§^	16 (1.0)
Fat (g day^−1^)	86 (17)^§§§^	316 (39)^†††^
Fat (g kg day^−1^)	1.3 (0.1)^§§§^	4.9 (0.4)^†††^
Fat (E%)	20 (0.2)^§§§^	80 (1.0)^†††^
EEE (kcal day^−1^)	1648 (472)	1187 (222)^†^
EA (kcal kg FFM day^−1^)	39 (1)	40 (1)
Restoration period: both groups – HCHO diet		
Energy intake (kJ day^−1^)	15777 (3392)	15310 (1868)
Energy intake (kJ kg day^−1^)	241 (32)	235 (11)
CHO (g day^−1^)	624 (135	601 (72)
CHO (g kg day^−1^)	9.5 (1.3)	9.2 (0.4)
CHO (E%)	66 (0.7)	66 (0.4)
Protein (g day^−1^)	139 (31)	137 (17)
Protein (g kg day^−1^)	2.1 (0.3)	2.1 (0.1)
Protein (E%)	15 (0.2)	15 (0.2)
Fat (g day^−1^)	84 (18)	82 (11)
Fat (g kg day^−1^)	1.3 (0.2)	1.3 (0.1)
Fat (E%)	20 (0.7)	20 (0.3)
EEE (kcal day^−1^)	1533 (693)	1358 (232)
EA (kcal kg FFM day^−1^)	39 (2)	39 (1)

Data are means (SD). ^**^ *P *< 0.01, ^***^ *P *< 0.001; significant within‐group difference between baseline and adaptation. ^†^ *P *< 0.05, ^†††^ *P *< 0.001; significant within‐group difference between adaptation and restoration. ^‡^ *P *< 0.05, ^‡‡^ *P *< 0.01; significant within‐group difference between baseline and restoration. ^§^ *P *< 0.05, ^§§§^ *P *< 0.001; significant difference between HCHO and LCHF. HCHO, high energy/high carbohydrate availability diet; LCHF, ketogenic low carbohydrate high fat diet.

**Table 3:** Results of graded economy test and maximal aerobic capacity prior to and following 5–6 days’ adaptation to ketogenic LCHF diet or HCHO diet in elite race walkers undertaking endurance training

	HCHO (*n* = 6)					LCHF (*n* = 7)				
	S1	S2	S3	S4	Max	S1	S2	S3	S4	Max
Body mass (kg)										
Baseline	66.2 (7.8)					66.2 (8.1)				
Adaptation	65.5 (7.9)					64.9 (8.2)^†^				
Respiratory exchange ratio										
Baseline	0.90 (0.02)	0.94 (0.01)	0.97 (0.02)	1.01 (0.02)	1.12 (0.03)	0.92 (0.02)	0.95 (0.02)	0.98 (0.02)	1.02 (0.03)	1.11 (0.04)
Adaptation	0.87 (0.03)	0.90 (0.03)	0.94 (0.04)	0.97 (0.04)	1.09 (0.05)	0.71 (0.03)^‡^	0.75 (0.01)^‡^	0.79 (0.03)^‡^	0.86 (0.04)^‡^	0.97 (0.05)^‡^
${\dot{V}}_{O_{2}}$ (L min^−1^)										
Baseline	2.78 (0.34)	3.07 (0.38)	3.43 (0.46)	3.70 (0.50)	4.23 (0.51)	2.82 (0.42)	3.14 (0.45)	3.56 (0.48)	3.93 (0.51)	4.46 (0.56)
Adaptation	2.71 (0.41)	3.00 (0.41)	3.41 (0.46)	3.64 (0.55)	4.28 (0.51)	2.92 (0.53)^†^	3.28 (0.53)^†^	3.66 (0.59)^†^	4.02 (0.59)^†^	4.52 (0.61)
Heart rate (BPM)										
Baseline	133 (13)	146 (10)	157 (10)	167 (11)	180 (13)	143 (7)	156 (5)	168 (7)	178 (7)	192 (8)
Adaptation	131 (13)	145 (10)	156 (9)	164 (10)	177 (9)	148 (8)	160 (10)	172 (9)	180 (8)	192 (10)
RPE										
Baseline	9.0 (2.5)	11.35 (1.9)	13.3 (1.2)	15.3 (1.2)		9.4 (2.1)	10.6 (2.4)	13.0 (2.1)	15.3 (1.7)	
Adaptation	9.2 (1.6)	11.3 (1.2)	12.5 (1.4)	14.2 (1.9)		10.9 (1.8)^‡^	12.6 (1.9)^‡^	14.3 (2.9)^‡^	15.7 (2.3)	

Data are mean (SD). Significant differences within group relative to Baseline: ^†^ *P *< 0.005, ^‡^ *P *< 0.0001. HCHO, high energy/high carbohydrate availability; LCHF, ketogenic low carbohydrate high fat.

## Results

### Diet and training compliance

All athletes demonstrated compliance to their assigned dietary treatment and the monitoring of their food intake and training sessions. Table 2 summarises the results of the assessment of actual dietary intake, with the summary of mean daily intakes allowing the display of overall differences between diets. Assessments of exercise energy expenditure (EEE) showed a daily mean of ∼1600 kcal (6720 kJ) for HCHO group across all phases. Overall EEE was not different between groups, but the LCHF group incurred a reduced expenditure during the intervention compared with Baseline (*P *= 0.008) and Restoration (*P *= 0.18). There were no significant differences between groups for energy and macronutrient intake characteristics for the Baseline and Restoration phases (see Table 2), and each group was able to achieve the main targets for these phases: EA of ∼40 kcal (168 kJ) kg^−1^ FFM day^−1^, protein intake of ∼2 g kg^−1^ BM day^−1^, and CHO intake of 9–9.5 g kg^−1^ BM day^−1^ contributing ∼65% of energy. During the intervention phase, protein intake was maintained across both groups at the target of ∼2.2 g kg^−1^ BM day^−1^. The LCHF group achieved the targets of their dietary prescription, consuming <5% of energy and <0.5 g kg^−1^ as CHO and 80% of energy from fat.

### ${\dot{V}}_{O_{2}\mathrm{peak}}$ and economy testing

All race walkers participated in the graded economy and ${\dot{V}}_{O_{2}\mathrm{peak}}$ test protocols. The results of these tests are summarised in Table 3 (BM, RER, absolute ${\dot{V}}_{O_{2}}$ (L min^−1^), HR and RPE data), Fig. 2 (relative ${\dot{V}}_{O_{2}}$ (ml min^−1^ kg^−1^) and calculated substrate utilisation) and Fig. 3 (blood metabolites). There were no differences between groups at baseline (*P *= 0.419) or across the intervention period in absolute ${\dot{V}}_{O_{2}\mathrm{peak}}$ (HCHO, *P *= 0.315; LCHF, *P *= 0.493). Both groups displayed an increase in RER, absolute ${\dot{V}}_{O_{2}}$ (L min^−1^), HR and RPE associated with the increase in exercise intensity across all four stages of both economy tests (all *P *< 0.0001). Within the LCHF group, there was a significant effect of the dietary intervention (Baseline *vs*. Adaptation) on metabolic cost and perceived effort, with changes to absolute ${\dot{V}}_{O_{2}}$ (L min^−1^) (Stages 1–4 higher, *P *< 0.005; ${\dot{V}}_{O_{2}\mathrm{peak}}$ *P *= 0.493), RER (all stages lower following adaptation, *P *< 0.0001) and RPE (Stages 1–3 higher, *P *< 0.0001; Stage 4, *P *= 0.149). In contrast, there were no diet‐associated effects within the HCHO group for ${\dot{V}}_{O_{2}}$ (L min^−1^), RER, HR or RPE (Table 3).

These findings are supported by the change observed in relative ${\dot{V}}_{O_{2}}$ (ml min^−1^ kg^−1^, Fig. 2*A*) and rates of CHO and fat oxidation (Fig. 2*B* and *C*). While there was a main effect for stage associated with increasing exercise intensity in the HCHO diet, no effect of diet was detected on relative ${\dot{V}}_{O_{2}}$ (ml min^−1^ kg^−1^, *P *= 0.812) or fat oxidation rates (*P *= 0.139). However, the rate of CHO oxidation was lower across all four stages (*P *= 0.0004 and 0.0005 for Stages 1 and 3, *P *< 0.0001 for Stages 2 and 4). Within the LCHF diet, relative ${\dot{V}}_{O_{2}}$ (ml min^−1^ kg^−1^) was higher across all submaximal stages following dietary adaptation (all *P *< 0.0001), indicating a decrease in exercise economy relative to Baseline when walking at the same absolute speed, with the magnitude of the increase being ∼6% and 4% at Stages 2 and 4, respectively. This increase in O_2_ consumption was associated with the increased reliance on fat as a metabolic substrate, as fat oxidation rates increased across all four stages (*P *< 0.0001), peaking at 1.43 g min^−1^ in the first stage (11–12 km h^−1^), while CHO oxidation displayed a reciprocal decrease across all stages (*P *< 0.0001).

Figure 3 summarises circulating concentrations of metabolites (glucose, lactate and ketones) obtained via capillary blood samples measured during the economy test, representing resting values 2 h following a standardised meal, concentrations at the end of each stage and at the conclusion of the incremental test. There was a main effect of stage on blood glucose concentrations, reflecting exercise intensity, but this was unaltered by adaptation to diet in either the HCHO (*P *= 0.694) or LCHF (*P *= 0.308) group (Fig. 3*A*). Blood lactate concentrations increased with exercise intensity across both dietary treatments (*P *< 0.0001), with similar patterns in both pre‐ and post‐treatment trials for the HCHO group (Fig. 3*B*). In contrast, blood lactate concentrations at the two highest intensity stages were suppressed following Adaptation in the LCHF group (Stage 3, *P *= 0.030; Stage 4, *P *= 0.048). Blood ketone levels in the HCHO group were maintained at low concentrations (0.0–0.1 mmol L^−1^), with no differences across the economy test for both pre‐ and post‐treatment. However, the LCHF group displayed significant increases in blood ketone concentrations following the dietary intervention (*P *= 0.008), which were highest at rest (1.2 (0.79) mmol L^−1^). Despite declining over the subsequent economy stages, ketone concentrations remained elevated at all stages in comparison to the Baseline (all *P *< 0.0001).

### Twenty‐five kilometre walk

All athletes performed the three hybrid laboratory‐field test 25 km walks, which are summarised in Table 4 (BM, RER, absolute ${\dot{V}}_{O_{2}}$ (L min^−1^), HR and RPE data), Fig. 4 (relative ${\dot{V}}_{O_{2}}$ (ml min^−1^ kg^−1^) and substrate utilisation) and Fig. 5 (blood metabolites). In the HCHO group, there were no differences in time to complete the 25 km walk across the study (data not shown, ∼2:01 h, all *P *> 0.999), while BM decreased by an average of 0.96 (0.44) kg over each of the three ∼2 h training walks (*P *< 0.0001). The LCHF group followed a similar trend for BM changes in Walk 1 (0.92 (0.72) kg, *P *< 0.0001) and Walk 3 (0.69 (0.39) kg, *P *= 0.0002), but there was no significant change in post‐exercise body mass following Walk 2 (0.28 (0.45) kg, *P *= 0.110). Walk 2 undertaken with the LCHF diet was slightly but significantly slower than the other walks (2:11:44 (0:09:26), *P *= 0.0016 *vs*. Walk 1, 2:04:14 (0:05:34), *P *= 0.0005 *vs*. Walk 3, 2:03:22 (0:04:02); Walk 1 *vs*. Walk 3, *P *= 0.953).

**Table 4:** Results of prolonged 25 km metabolic challenge prior to and following 5–6 days’ nutritional intervention and 5–6 days’ restoration period

	HCHO (*n* = 6)						LCHF (*n* = 7)				
	1 km	7 km	13 km	19 km	25 km		1 km	7 km	13 km	19 km	25 km
Body mass (kg)											
Week 1^†^	67.04 (8.11)				65.82 (7.79)^***^	Week 1^‡^	66.87 (8.29)				65.95 (8.05)^***^
Week 2	66.37 (8.30)				65.49 (7.84)^***^	Week 2	64.72 (8.18)				64.44 (8.16)
Week 3	65.65 (7.65)				64.86 (7.51)^***^	Week 3^‡‡^	66.16 (8.30)				65.47 (8.18)^**^
Respiratory exchange ratio											
Week 1	0.92 (0.02)	0.90 (0.01)	0.90 (0.02)	0.87 (0.02)	0.87 (0.02)	Week 1^‡^	0.94 (0.02)	0.92 (0.02)	0.92 (0.03)	0.89 (0.02)	0.87 (0.04)
Week 2	0.91 (0.02)	0.88 (0.01)	0.88 (0.02)	0.87 (0.02)	0.86 (0.03)	Week 2	0.77 (0.03)	0.75 (0.02)	0.76 (0.02)	0.77 (0.03)	0.77 (0.03)
Week 3^‡^	0.94 (0.02)	0.90 (0.02)	0.89 (0.03)	0.87 (0.04)	0.87 (0.03)	Week 3^‡^	0.95 (0.03)	0.93 (0.03)	0.93 (0.03)	0.90 (0.03)	0.89 (0.02)
${\dot{V}}_{O_{2}}$ (L min^−1^)											
Week 1	3.13 (0.48)	3.16 (0.48)	3.13 (0.52)	3.24 (0.50)	3.26 (0.47)	Week 1	3.30 (0.39)	3.37 (0.32)	3.20 (0.52)	3.33 (0.42)	3.37 (0.40)
Week 2	3.16 (0.39)	3.16 (0.47)	3.19 (0.50)	3.25 (0.45)	3.34 (0.49)	Week 2	3.39 (0.43)	3.42 (0.40)	3.42 (0.39)	3.41 (0.43)	3.45 (0.40)
Week 3^‡^	3.07 (0.48)	3.05 (0.45)	3.06 (0.47)	3.10 (0.48)	3.16 (0.42)	Week 3^‡^	3.15 (0.36)	3.18 (0.40)	3.25 (0.36)	3.26 (0.40)	3.34 (0.38)
Heart rate (BPM)											
Week 1	132 (10)	150 (8)	152 (5)	153 (5)	158 (7)	Week 1^‡^	148 (5)	154 (4)	154 (6)	157 (6)	159 (7)
Week 2	137 (7)	149 (8)	150 (8)	150 (6)	155 (7)	Week 2	152 (12)	161 (9)	165 (6)	165 (6)	170 (9)
Week 3	135 (8)	146 (15)	149 (9)	149 (8)	153 (8)	Week 3^‡‡^	148 (8)	151 (5)	151 (8)	151 (9)	157 (11)
RPE											
Week 1	10.3 (2.6)	11.0 (2.1)	12.2 (1.7)	13.2 (1.9)	14.2 (1.6)	Week 1^‡‡^	11.3 (1.3)	11.9 (1.6)	13.0 (1.4)	13.4 (1.4)	14.1 (2.5)
Week 2	9.6 (3.0)	11.9 (1.6)	12.5 (1.6)	13.8 (1.5)	14.1 (2.5)	Week 2	13.6 (2.4)	14.6 (2.1)	14.4 (1.8)	15.6 (2.8)	16.7 (2.4)
Week 3	9.9 (1.7)	11.3 (1.6)	12.0 (0.9)	12.8 (0.8)	13.0 (1.0)	Week 3^‡‡^	11.3 (1.5)	12.14 (1.2)	12.7 (1.0)	13.1 (1.5)	13.7 (1.6)

Data are means (SD). Significant differences within group relative to 1 km: *P < 0.05, **P < 0.005, ***P < 0.0001. Significant differences within group relative to Week 3: ^†^ *P *< 0.005. Significant differences within group relative to Week 2: ^‡^ *P *< 0.05, ^‡‡^ *P *< 0.005, ^‡^ *P *< 0.0001.

Due to the prolonged nature of the exercise, both groups displayed a decrease in RER over the course of the 25 km walks (Table 4, main effect of distance, *P *< 0.0001), which was associated with a significant increase in both absolute and relative ${\dot{V}}_{O_{2}}$ (Table 4 and Fig. 4*A* and *D*), respectively) and a gradual reduction in CHO (Fig. 4*B*, *P *= 0.001; Fig. 4*E*, *P *= 0.0001) and increase in fat oxidation (Fig. 4*C*, *P *< 0.0001; Fig. 4*F*, *P *< 0.0001). Within the HCHO group, there were no differences between Walks 1–3 in relative ${\dot{V}}_{O_{2}}$ (Fig. 4*A*, *P *= 0.113) or CHO oxidation (Fig. 4*B*, *P *= 0.258); however, a main effect for testing block was detected for fat oxidation (Fig. 4*C*, *P *= 0.003), with Walk 2 (Adaptation) displaying higher overall rates compared to Walk 1 (Baseline, *P *= 0.03) and 3 (Restoration, *P *= 0.003). Similarly, RPE (*P *< 0.0001) and HR (*P *< 0.0001) increased across the test for the HCHO group, but this was not different between Walks 1–3 (*P *= 0.906).

Consistent with the intent of the dietary intervention, the LCHF group displayed substantially altered fuel utilisation in Walk 2, with fat oxidation being significantly increased in comparison to Walks 1 and 3 (*P *< 0.0001), and a reciprocal decrease in CHO oxidation (*P *< 0.0001). While HR (*P *< 0.0001) and RPE (*P *= 0.0005) increased across the session for all three walks in the LCHF group, this was more pronounced in the Adaptation test block (Table 4). When combined with the significant increase in the relative oxygen cost of walking in Walk 2 (Fig. 4*D*; *P *= 0.005 *vs*. Walk 1, *P *= 0.001 *vs*. Walk 3), this indicates an increase in the perceived effort and metabolic cost of exercise when relying on fat as the primary fuel. Critically, there were no differences in relative ${\dot{V}}_{O_{2}}$ (Fig. 4*D*, *P *= 0.999) or substrate utilisation (Fig. 4*E*, *P *< 0.999, Fig. 4*F*, *P *= 0.850) between Walk 1 and Walk 3, indicating these effects were transient and the restoration period was sufficient to restore metabolic flexibility.

Changes in whole‐body metabolism were supported by the changes in circulating substrate availability measured in capillary and venous blood samples (Fig. 5). There were no differences in blood glucose concentrations across testing blocks (Walks 1–3) in the HCHO group (*P *= 0.355), but there was a main effect for distance as blood glucose increased across the 25 km trial consistent with the continuous intake of CHO (*P *= 0.0009). In contrast, the LCHF group displayed a main effect for testing block (*P *< 0.0001), with blood glucose concentrations (Fig. 5*A*) increasing in Walk 1 (Baseline) and to a lesser extent in Walk 3 (Restoration, *P *= 0.03 *vs*. Walk 1), but decreasing in Walk 2 (Adaptation, *P *< 0.0001 vs Walk 1 and 3). There was a main effect observed for both distance (*P *= 0.0046) and test block (*P *< 0.0001) in blood lactate responses in the LCHF group (Fig. 5*B*), as there was a decrease across the trial in Walks 1 and 3, while lactate increased across Walk 2 (*P *= 0.0007 *vs*. Walk 1, *P *< 0.0001 *vs*. Walk 3). Similarly, there was a robust increase in circulating blood β‐hydroxybutyrate in the Walk 2 in the LCHF group (*P *= 0.0004 *vs*. Walk 1 and Walk 3), peaking at 1.7 (0.9) mmol L^−1^ towards the end of the trial, but there were no differences in the HCHO group. There was an increase in serum FFA concentrations across the duration of the walk (*P *< 0.0001) in both groups (Fig. 5*D*), but only the LCHF group displayed differences between testing blocks, with concentrations being significantly elevated at all time points in Walk 2 after the LCHF diet compared to Walks 1 and 3 (*P *< 0.0001).

### Pre‐race substrate utilisation

To determine the effects of the 1‐day CHO restoration on substrate utilisation during exercise, athletes in the LCHF group reported to the laboratory on the morning of the 10,000 m race in the Baseline and Adaptation testing blocks and repeated Stages 2 and 4 of the submaximal exercise economy test within their pre‐race warm‐up (Fig. 6). There were no differences in calculated substrate utilisation between economy test and pre‐race measurements in the Baseline testing block. Following the 6‐day adaptation to LCHF and a 1‐day HCHO diet designed to enhance skeletal muscle glycogen availability, fat oxidation rates at Stages 2 and 4 were reduced by 40% (*P *= 0.011) and 59% (*P *= 0.0002), respectively, relative to the economy test performed following LCHF adaptation (Fig. 6*B* and *D*), but remained elevated compared to the first pre‐race test performance performed at Baseline (Stage 2; *P *= 0.0008, Stage 4; *P *= 0.002). While CHO oxidation rates at Stages 2 and 4 were increased by 197% (*P *= 0.0015) and 51% (*P *= 0.0007) compared to the LCHF adaptation trial, they remained suppressed relative to the pre‐race test performed at Baseline (Stage 2, *P *= 0.002; Stage 4, *P *= 0.003) (Fig. 5*A* and *C*).

### 10,000 m race

Real world performance outcomes from these dietary interventions were tested by having athletes compete in WA sanctioned 10,000 m races. Environmental conditions for Baseline and Adaptation races, respectively, were 28.7 (1.2)°C and 22.8 (0.3)°C, 47.1 (3.5)% and 72.1 (1.2)% relative humidity, and wind speeds of 1.5 (0.4) m s^−1^ (with gusts up to 2.6 m s^−1^) and 1.2 (0.3) m s^−1^ (gusts up to 2 m s^−1^). Athletes raced competitively, with finishing times for the post‐intervention races representing 103 (3)% and 104 (8)% of the life‐time personal best times of walkers in the HCHO and LCHF groups, respectively, and four athletes recording times faster than their personal bests (three from the HCHO group under Baseline or Adaptation races and one from the LCHF group for Adaptation race). Performance results are presented in Fig. 7, with a summary of aerobic testing (Fig. 7 *A*), individual race results (Fig. 7 *B*) and difference in performances between races (Fig. 7 *C*). There was a significant interaction between diet and time for race results (*P *= 0.02), and a significant difference between performance changes between the two groups (*P *= 0.009). Despite the lack of a statistically significant (*P *= 0.222) change in maximum aerobic capacity (relative ${\dot{V}}_{O_{2}\mathrm{peak}}$, Fig. 7*A*), all six athletes in the HCHO group improved performance by an average of 5.7 (5.6)%. In contrast, 6 of 7 LCHF athletes displayed impaired performance with a mean race time that was 2.2 (3.4)% slower (Fig. 7*B*), despite a small but significant increase in relative ${\dot{V}}_{O_{2}\mathrm{peak}}$ (*P *= 0.025). The difference between changes in performance from Race 1 to Race 2 was significant (*P *= 0.009) (Fig. 7*C*).

## Discussion

This paper continues our series of investigations of the ketogenic LCHF diet in world class endurance athletes. The novel findings from this study are: (1) adaptations that substantially increase fat oxidation during exercise in response to the LCHF occur in elite athletes in as little as 5–6 days, reaching the same rates as observed in endurance athletes who have adhered to this diet for medium (3–4 weeks) and long‐term periods (>12 weeks); (2) muscle retooling to enhance fat oxidation with brief exposure to a ketogenic LCHF is maintained in the face of acute increases in muscle CHO availability, but is reversed by 5 days of chronic high CHO diet; (3) the economy of exercise at intensities relevant to real life endurance events is reduced by brief adaptation to a ketogenic LCHF when fat oxidation is maximised; and (4) strategies that attempt to integrate brief adaptation to a ketogenic LCHF diet with enhanced muscle CHO availability seem limited by the blunting of CHO oxidation and impaired performance of endurance events undertaken at high relative and absolute exercise intensities.

There is clear evidence from both intervention (Phinney *et al*. 1983; Burke *et al*. 2017; Shaw *et al*. 2019; 2020) and cross‐sectional studies (Volek *et al*. 2016; Webster *et al*. 2016) that adherence to a ketogenic LCHF diet leads to substantial (e.g. 200–250%) increases in capacity for fat oxidation during exercise, even in well‐trained endurance athletes who achieve this characteristic as an outcome of their training (Hawley *et al*. 2018). Mean fat oxidation rates of ∼1.5 g min^−1^ have been reported during protocols of graded (Volek *et al*. 2016) or prolonged sustained exercise (Burke *et al*. 2017) following adaptation to ketogenic LCHF diet. It should be noted that such rates reflect absolute workloads, and interaction of factors such as the athlete's size and maximal aerobic capacity; indeed some studies have reported rates approaching 2 g min^−1^ in elite athletes with larger BM and high ${\dot{V}}_{O_{2}\mathrm{peak}}$ characteristics (Burke *et al*. 2017). Meanwhile, the lower maximal fat oxidation rates of 1.1–1.4 g min^−1^ reported in other studies of ketogenic LCHF diets (Prins *et al*. 2019; Shaw *et al*. 2019; Burke *et al*. 2020) may reflect low BM or lower absolute aerobic capacity of the athlete (including females or individuals of lower calibre) or different exercise protocols, rather than incomplete adaptation. Therefore, it might be useful to report such values in terms of g kg^−1^ BM min^−1^ to at least take one likely co‐factor into account. In any case, the current study adds a significant piece to the current literature by showing that this core characteristic of keto adaptation can be achieved in a much shorter time frame than previously considered.

The time frame over which the LCHF diet achieves its putative benefits is controversial. Information sources underpinning the resurgence of interest in this diet identified that ‘if humans are given two or more weeks to adapt to a well‐formulated low carbohydrate diet, they can deliver equal or better endurance performance compared to the best high carbohydrate strategy’ (Volek & Phinney, 2012, p. 20). However, subsequent studies involving 3–4 weeks’ interventions were heavily criticised in the scientific literature and lay commentary as being too brief to allow the full advantages of the diet to occur (Burke *et al*. 2020). Notwithstanding the potential for other physiological or metabolic outcomes to occur over different time frames, our current study showed that within 5–6 days of adherence to a ketogenic LCHF diet, rates of fat oxidation during exercise reached a mean of ∼1.43 g min^−1^ during a graded economy test and a prolonged training session at speeds that are relevant to the 50 km race walking event. This is similar to the mean values reported in our first study (Burke *et al*. 2017) after a 3.5‐week period of keto adaptation in a similar cohort of elite male race walkers (∼1.57 g min^−1^ during the ∼2 h race walking session). Moreover, it is equivalent to the fat values (∼1.54 g min^−1^) reported by Volek and colleagues (2016) from highly competitive ultra‐distance runners and triathletes who had self‐selected to follow a ketogenic LCHF diet for an average of 20 months. Other metabolic features associated with 3.5 weeks of keto adaptation (Burke *et al*. 2017, 2020) were also observed after 5–6 days; blood glucose was maintained at lower, albeit euglycaemic, concentrations during exercise while βHB was elevated above 1 mmol L^−1^ at rest and during exercise.

Our results are not entirely unexpected. Indeed, a robust literature on non‐ketogenic versions of LCHF (∼2–2.5 g kg BM^−1^ day^−1^ CHO, ∼65% energy from fat) from nearly two decades ago showed that adaptations which significantly and robustly up‐regulate capacity for fat oxidation occur in a relatively brief period. A study of the time course of changes in metabolism, substrate utilisation and muscle enzyme activities in endurance‐trained athletes following a non‐ketogenic LCHF diet reported substantial increases in exercise fat oxidation after 5 days that were not further enhanced with longer periods of exposure (Goedecke *et al*. 1999). These findings provoked a series of studies which confirmed that rates of fat oxidation during exercise were doubled after 5 days of exposure to a non‐ketogenic LCHF diet (Burke *et al*. 2000; Carey *et al*. 2001, 2002; Stepto *et al*. 2002). Biopsy‐derived investigations of muscle within these studies provided evidence of coordinated retooling to enhance capacity for fat utilisation within this time frame, with reported increases in intramuscular triglycerides (Yeo *et al*. 2008), the activities of hormone‐sensitive lipase (Stellingwerff *et al*. 2006) and carnitine palmitoyl transferase (Goedecke *et al*. 1999) as well as the protein expression of lipid transporters fatty acid translocase FAT/CD36 (Cameron‐Smith *et al*. 2003). Although studies of adaptation to ketogenic LCHF diets have failed to directly investigate the mechanism(s) underpinning enhanced fat utilisation, it is likely that similar up‐regulation of fat availability, mobilisation, transport and mitochondrial uptake within the muscle is responsible.

Data from the current study mirror another finding from these earlier investigations of the non‐ketogenic LCHF diet, that muscle retooling is maintained for at least 24 h of reintroduction to HCHO availability and muscle glycogen restoration. The inability to undertake invasive procedures with world class athletes prevented us from taking muscle biopsies to measure pre‐race muscle glycogen stores. However, we strictly replicated dietary control and exercise conditions for the 24 h prior to each race, including a pre‐race breakfast. This provides some confidence that similar restoration of muscle (and liver) glycogen should have occurred across trials and between groups. For example, in our previous studies of non‐ketogenic LCHF diets, we found that 24 h of a high CHO diet and tapered training undertaken after 5 days of training with either HCHO availability or a non‐ketogenic LCHF diet allowed restoration and equalisation of muscle glycogen content (Burke *et al*. 2000). The current investigation involved a real‐life endurance race to measure the effects of keto adaptation plus acute enhancement of whole‐body CHO availability on performance. Nevertheless, we were able to overcome the practical challenges typically involved in collecting mechanistic data under field conditions by having the LCHF‐adapted athletes undertake a portion of their pre‐race warm up in the laboratory. We were able to examine substrate use (Fig. 6) and exercise economy at two chosen treadmill speeds during a graded economy test and the pre‐race warm up, 24 h apart. In the case of Race 1, we found tight replication of substrate use at the Stage 2 and Stage 4 treadmill speeds which relate to the pace required for the 50 km and 20 km race walking events on the current Olympic program, respectively. During the Race 2 warm‐up, acute restoration of CHO availability increased rates of CHO oxidation and reduced the contribution of fat oxidation to energy production compared with the previous day's economy test undertaken under full keto adaptation conditions. However, this represented an intermediary shift in substrate use, with CHO oxidation failing to be fully restored to Baseline rates. Indeed, rates of CHO oxidation reached only 61% and 78% of the values seen under the same conditions as Race 1 for Stages 2 and 4, respectively.

Our previous studies of 5–6 days’ adaptation to a non‐ketogenic LCHF and 24 h restoration of CHO availability (Burke *et al*. 2000; Carey *et al*. 2001, 2002) produced strikingly similar results. Here we found a hierarchical demonstration of substrate use during exercise according to the degree of restoration of CHO availability, with restoration of muscle glycogen plus a pre‐race CHO‐rich meal and CHO intake during exercise achieving greater rates of CHO oxidation than the same exercise task undertaken with glycogen restoration but fasted exercising conditions (Burke *et al*. 2002). Nevertheless, even when all strategies to optimise CHO availability were combined, prior adaptation to the high‐fat diet was associated with a relative suppression of rates of CHO oxidation and an increased reliance on fat utilisation. Investigation of muscle substrate use both by direct (biopsy‐derived) and by indirect tracer techniques demonstrate a down‐regulation of endogenous (i.e. muscle glycogen) rather than exogenous CHO use (Burke *et al*. 2000, 2002; Carey *et al*. 2001). Mechanisms for what we had initially considered to be ‘glycogen sparing’ but subsequently recognised as ‘glycogen impairment’ include a decrease in glycogenolysis and a reduction in the active form of pyruvate dehydrogenase (Stellingwerff *et al*. 2006).

Examination of changes in CHO metabolism in keto‐adapted athletes is less well developed, although there is consistent evidence of a reduction in muscle glycogen oxidation (Phinney *et al*. 1983, Volek *et al*. 2016, Webster *et al*. 2016). Phinney and co‐workers (1983) reported that glycogen utilisation was reduced 4‐fold, and blood glucose utilisation, 3‐fold, during moderate intensity (62–64% ${\dot{V}}_{O_{2}\mathrm{peak}}$) cycling after a 4‐week ketogenic LCHF diet. Meanwhile, Webster *et al*. (2016) reported lower rates of muscle glycogen oxidation and hepatic glucose production at rest and during cycling at ∼70% ${\dot{V}}_{O_{2}\mathrm{peak}}$ in keto‐adapted ultra‐endurance athletes compared to a cohort consuming higher CHO diets. They hypothesised that this represents reduced liver glycogen breakdown without compensation by an absolute increase in gluconeogenesis. Furthermore, it has recently been shown that athletes who adhere to a ketogenic‐LCHF diet for longer periods (i.e. >6 months) display impaired glucose tolerance during an oral glucose tolerance test as a result of decreased GLUT4 and insulin receptor substrate 1 (IRS1) protein content (Webster *et al*. 2020). Until future studies investigate tissue metabolite and enzyme activities underpinning changes in muscle and whole‐body CHO metabolism, we can only speculate whether keto adaptation produces differences to the changes seen in response to a non‐ketogenic LCHF diet. Although our current study does not provide such information, it contributes new knowledge by showing that gross changes in substrate utilisation and metabolism during exercise in response to keto adaptation are reversed by 5 days of reintroduction of a HCHO diet. Our analysis of data from the 25 km training sessions shows that characteristics that were perturbed during the Adaptation session after 5–6 days of adherence to the LCHF diet (e.g. blood metabolites (Fig. 6), rates of CHO and fat oxidation (Fig. 5), heart rates and perception of effort (Table 4), and chosen pace for the ‘outside portion’ of the training session all had reverted to Baseline values after 5 days of return to HCHO. This demonstrates that de‐adaptation probably follow a similar time course to adaptation; although our data cannot inform the processes that occur between 1 and 5 days of each exposure, it seems that robust modifications are in place by this latter time point. Furthermore, it is likely that the changes in metabolic cost and perceived effort of exercise are likely related to the immediate patterns of substrate use rather than reflecting a legacy of previous well‐being and training capacity.

We approached the most important metric in our study, athletic performance, using the authenticity of real‐life races. The 10,000 m track race was chosen because it reduces the variability in environmental and course conditions encountered in road events; it can be raced at peak level on two occasions over the duration of the study; the pace is relevant to the current Olympic race walking events (i.e. it is similar to 20 km event speeds and recognises the pace sustained over critical phases towards the end of the 50 km event; Huebsch 2017) and because we have gained robust and reproducible outcomes from our previous studies (Burke *et al*. 2017, 2020). Even though this event is not glycogen limited, its relevance to longer races is exemplified by the strong correlation between 10 km personal best times and marathon performance in specialist distance runners (Noakes *et al*. 1990). The performance changes from Race 1 to Race 2 in the current study were significantly different and manifested as a uniform and significant improvement in race times in the HCHO group and a strong trend to performance impairment in the LCHF group, with only one participant recording a faster race outcome in Race 2 following the LCHF adaptation/enhanced CHO availability intervention. We acknowledge that different environmental conditions and the ‘training camp effect’ (Saunders *et al*. 2010) potentially contributed to the performance changes from Race 1 to Race 2. However, these should have affected both groups equally, and favourably. The ∼8% difference in performance changes between the HCHO and LCHF groups was strikingly similar to our previous findings (Burke *et al*. 2017, 2020), providing additional confidence around these results.

We have previously attributed at least part of the performance impairment with the LCHF diet to a reduction in exercise economy (increased oxygen cost of exercise), noting that a high exercise economy is a hallmark of most elite endurance athletes (Joyner *et al*. 2020) and a target of a variety of strategies to increase endurance performance (Santos‐Concejero *et al*. 2020). Here, it is underpinned by an understanding of the stoichiochemistry of the pathways of CHO and fat oxidation (for review, see Burke, 2021). However, other laboratories which have confirmed the loss of economy associated with adaptation to ketogenic LCHF diets, particularly at exercise intensities >75% ${\dot{V}}_{O_{2}\mathrm{peak}}$, have suggested that it is greater than can be accounted for via this explanation (Shaw *et al*. 2019) and other factors may be in play, including a change in the oral microbiome and downstream changes in the enterosalivary nitrate–nitric oxide pathway (Murtaza *et al*. 2019 or uncoupling of mitochondrial respiration (Leckey *et al*. 2018).

The results of our study suggest that acute restoration of muscle CHO availability is unable to rescue the impaired performance of endurance exercise undertaken at high relative and absolute intensities otherwise associated with the ketogenic LCHF diet (Burke *et al*. 2017, 2020). A similar outcome was reported by Havemann *et al*. (2006) in relation to short‐term exposure to a non‐ketogenic LCHF diet followed by strategies to optimise endogenous CHO availability during a performance trial (glycogen loading, pre‐race CHO‐rich meal and CHO intake during a prolonged endurance event). In this study, well‐trained subjects completed a 100 km cycling time trial with a crossover application of the fat‐adaptation, glycogen‐restoration strategy or a chronic HCHO diet. While overall time trial showed a non‐significant trend (3 min 43 s) towards faster time in the HCHO trial, the effects on performance were amplified with higher intensity exercise in the form of sprints integrated within the ‘race’. Although there was no significant effect on 4 km sprints performance at ∼80% of peak workload (*W*_peak_), the power output during 1 km sprints at ∼90% *W*_peak_ was significantly reduced with LCHF+HCHO, in association with markers suggestive of increased sympathetic drive and muscle recruitment (Havemann *et al*. 2006). Even those subjects who recorded a faster 100 km time showed reduced capacity in the 1 km sprints. The authors theorised that an impairment of CHO oxidation secondary to fat adaptation prevented the contribution of this important pathway to the fuel cost of the high‐intensity workloads.

Other strategies to periodise keto adaptation and restored CHO availability for an enhanced performance outcome have been studied (Webster *et al*. 2018; Burke *et al*. 2020) or observed in the practices of elite athletes (Woo & Olonan 2019). In a previous investigation (Burke *et al*. 2020), we were unable to find any evidence that periodising LCHF using a similar model to altitude training (e.g. 3.5 weeks’ adaptation to LCHF followed by 2.5 weeks’ return to taper and race preparation with high CHO availability) left a legacy of enhanced metabolism and performance in a 20 km race walking event (∼80 min duration). Indeed, based on the results of the current study, the gross effects of the LCHF on exercise fuel utilisation would have been washed out during this period. Meanwhile, a case history (Webster *et al*. 2018) in which a highly successful ultra‐endurance triathlete reintroduced exogenous CHO availability before or during a series of performance tasks reported a 2.8% improvement in 20 km cycling TT time, no change in 30 s sprint power, a small improvement in 4 min sprint power (1.6%), and a small reduction in 100 km TT time (1.1%). The intervention included the reintroduction of exogenous CHO availability (60 g h^−1^) during eight training sessions undertaken at higher intensity over a 3‐week period as well as CHO support immediately before or during the performance tasks. The authors concluded that this was ‘likely beneficial & during high‐intensity, endurance‐type exercise (4–30 min) but likely did not benefit short‐sprint or prolonged endurance performance.’ Exogenous CHO availability appeared to have a small effect on fat utilisation during the 100 km TT but increased rates of CHO oxidation (Webster *et al*. 2018). However, the mechanism of performance benefits observed in the higher intensity exercise could not be distinguished between alterations in fuel use, the effects of enhanced training support, or central nervous system effects of CHO mouth rinsing during the performance trials (Burke & Maughan, 2015). Indeed, others have noted that exposure of CHO to oral receptors enhances performance of higher intensity endurance exercise when endogenous CHO availability is low, with an enhanced central motor drive potentially being the main influencing mechanism (Ataide‐Silva *et al*. 2016). Of course, since this case history did not include strategies to increase endogenous CHO stores, or to compare outcomes on a fully CHO‐supported training and performance protocol, it cannot provide information about optimal dietary support for various types of exercise. Together, the available literature suggests that there are advantages of high CHO availability for higher intensity endurance tasks, but that it is difficult to fully integrate the maximal effects of keto adaptation and high CHO availability on performance.

In conclusion, we have shown that in highly trained endurance athletes, high rates of fat oxidation occur with as little as 5–6 days of adaptation to a ketogenic LCHF diet but reduce the economy of exercise at intensities relevant to real life endurance events, even when CHO availability is increased to provide an additional substrate. This adds to previous studies of periodisation of CHO availability with a non‐ketogenic LCHF diet, with novel elements including the use of elite athletes, rigorous implementation of dietary control with standardisation of energy availability, the measurement of performance via a real‐life sporting event, and the focus on higher exercise intensities that are critical for the success of high performance endurance athletes. We acknowledge some potential limitations of the study design; non‐random allocation to treatment to enhance placebo effects, differences *between* subjects in terms of pre‐race warm up and supplement use (although standardised *within* subjects for both races), and the possibility that the acute effects on race results were muddied by a chronic effect of the LCHF on training outcomes. Nevertheless, we feel that the majority of these methodological limitations were justified due to their ability to strengthen the real‐world application of results. Our findings suggest that muscle retooling to increase fat utilisation is maintained in the face of acute increases in muscle CHO availability, and appears to blunt capacity for muscle CHO oxidation. Muscle adaptations are reversed by reintroduction of the chronic high CHO diet in a similar time frame. Strategies that attempt to integrate brief adaptation to a ketogenic LCHF diet with enhanced muscle CHO availability seem limited by the blunting of CHO oxidation and continue to impair performance of endurance events undertaken at high relative and absolute exercise intensities. Further work to periodise or integrate the benefits of keto adaptation and high CHO availability is required, but it appears that maximal capacity for each oxidative pathway may not be simultaneously available. Therefore, endurance athletes who are contemplating the use of LCHF should undertake an audit of event *characteristics* and personal experiences to evaluate the importance of maximal rates of CHO oxidation to their goals: balancing the risk of impaired performance of higher intensity exercise with the potential benefits of having extended fuel stores for more moderate intensity exercise in the scenario of an unavoidable depletion of carbohydrate stores.

## Additional information

### Competing interests

None of the authors of this paper has a competing interest.

### Author contributions

This study was conducted at the Australian Institute of Sport, Canberra, Australia. Conception and design of the experiments was undertaken by L.M.B., J.W., I.A.H., A.K.A.M., M.L.R.R. and A.P.S. Collection, assembly, analysis and interpretation of data was undertaken by L.M.B., J.W., I.A.H., M.L.R.R., N.T., S.G.F., R.H., A.K.A.M., A.M.W. and A.P.S. Drafting the article or revising it critically for important intellectual content was undertaken by L.M.B. and JW. All authors have read and approved the final version of this manuscript and agree to be accountable for all aspects of the work in ensuring that questions related to the accuracy or integrity of any part of the work are appropriately investigated and resolved. All persons designated as authors qualify for authorship, and all those who qualify for authorship are listed.

### Funding

This study was funded by a Grant from Australian Catholic University Research Fund (ACURF, 2017000034). J.W. is supported by a Natural Sciences and Engineering Research Council of Canada (NSERC) Postdoctoral Fellowship.

## Supporting information


**Statistical Summary Document**

## Data Availability

The data that support the findings of this study are available from the corresponding author upon reasonable request.
